# Associations between albumin, globulin, albumin to globulin ratio and muscle mass in adults: results from the national health and nutrition examination survey 2011–2014

**DOI:** 10.1186/s12877-022-03094-4

**Published:** 2022-05-02

**Authors:** Zhi Chen, Chenyang Song, Zhipeng Yao, Jun Sun, Wenge Liu

**Affiliations:** 1 Department of Orthopedic Surgery, Fujian Medical University Union Hospital, Fuzhou, 350001 Fujian China; 2 Department of Emergency, Zhaotong Traditional Chinese Medicine Hospital, Zhaotong, 657000 Yunnan China

**Keywords:** Albumin, Globulin, Albumin to globulin ratio, Chronic inflammation, Skeletal muscle

## Abstract

**Introduction:**

Chronic inflammation and malnutrition play important roles in muscle loss. Although albumin, globulin and albumin to globulin ratio (AGR) are considered to be useful inflammatory-nutritional biomarkers, their relationship with muscle mass remain unclear. This study aimed to investigate the relationship between them in adults.

**Methods:**

We utilized data from the National Health and Nutrition Examination Survey (NHANES) 2011–2014 for analysis. Data on albumin, globulin, appendicular skeletal muscle mass, body mass index (BMI) and potential confounders (sociodemographic characteristics, medical conditions, laboratory parameters) were extracted and analyzed. We conducted multivariate linear regression models and smooth curve fittings to investigate the association between albumin, globulin, AGR and muscle mass. Subgroup analysis based on gender and muscle mass were performed.

**Results:**

A total of 4110 participants were included, there were 294 participants with low muscle mass (LMM) and 3816 participants with normal muscle mass (NMM). LMM individuals were older, had greater prevalence of diabetes, higher BMI, globulin and triglycerides, lower albumin and AGR. Albumin was positively correlated to muscle mass in men, but negatively correlated with muscle mass in women. There were negative association between globulin and muscle mass, and positive association between AGR and muscle mass among men, but no significant associations were detected among women. Moreover, a linear relationship between albumin, globulin and muscle mass, as well as a non-linear relationship between AGR and muscle mass in men were identified.

**Conclusions:**

The relationships between albumin, globulin, AGR and muscle mass were sex-specific. We speculate these indicators may be useful in assessing muscle mass in men.

**Supplementary Information:**

The online version contains supplementary material available at 10.1186/s12877-022-03094-4.

## Introduction

With the global population aging, the incidence of sarcopenia continues to rise, literatures reported the prevalence varied from 11 to 50% in people aged over 80 years old [1, 2]. A progressive decrease of skeletal muscle mass is the main feature of sarcopenia, which leads to poor metal health, increased morbidity rate, decreased physical ability, and declined quality of life [3–5]. Acknowledging the high prevalence and harmful impacts of muscle loss, more and more researches have been conducted to explore its pathogenesis, but the results remain incomplete and controversial [6].

The decrease of muscle mass was once considered to be the result of physical inactivity, malnutrition, neurodegeneration, and impaired hormone secretion [7]. Only recently have scholars started to investigate the links between age-related chronic low-grade inflammation and muscle loss [8]. A growing number of studies have demonstrated that individuals with low muscle mass have an elevated inflammatory marker profile [6, 9], which was believed to play an important role in the process of muscle decline [10]. Albumin can reflect not only the nutritional but also the inflammatory status [11]. Globulin consists of many proteins associated with inflammation, increases during the inflammatory process. The AGR takes into account both albumin and globulin to give a more accurate indication of the body’s nutritional and inflammatory status [12]. To date, whether there are any associations between these indicators and muscle mass remain largely unknow. Therefore, we conducted this study to investigate the relationships between albumin, globulin, AGR and muscle mass.

## Methods

### Research design and study population

The National Health and Nutrition Examination Survey is a cross-sectional survey, which provides a wealth of information on the nutrition and health of the general population in United States using a multistage, complex clustered, probability sampling design [13]. The NHANES protocols were approved by the National Center for Health Statistics ethics review board, and informed consent forms were obtained from all participants [14]. The survey data are free available for researchers worldwide, and operational instructions are available on the CDC website.

**Fig. 1:**
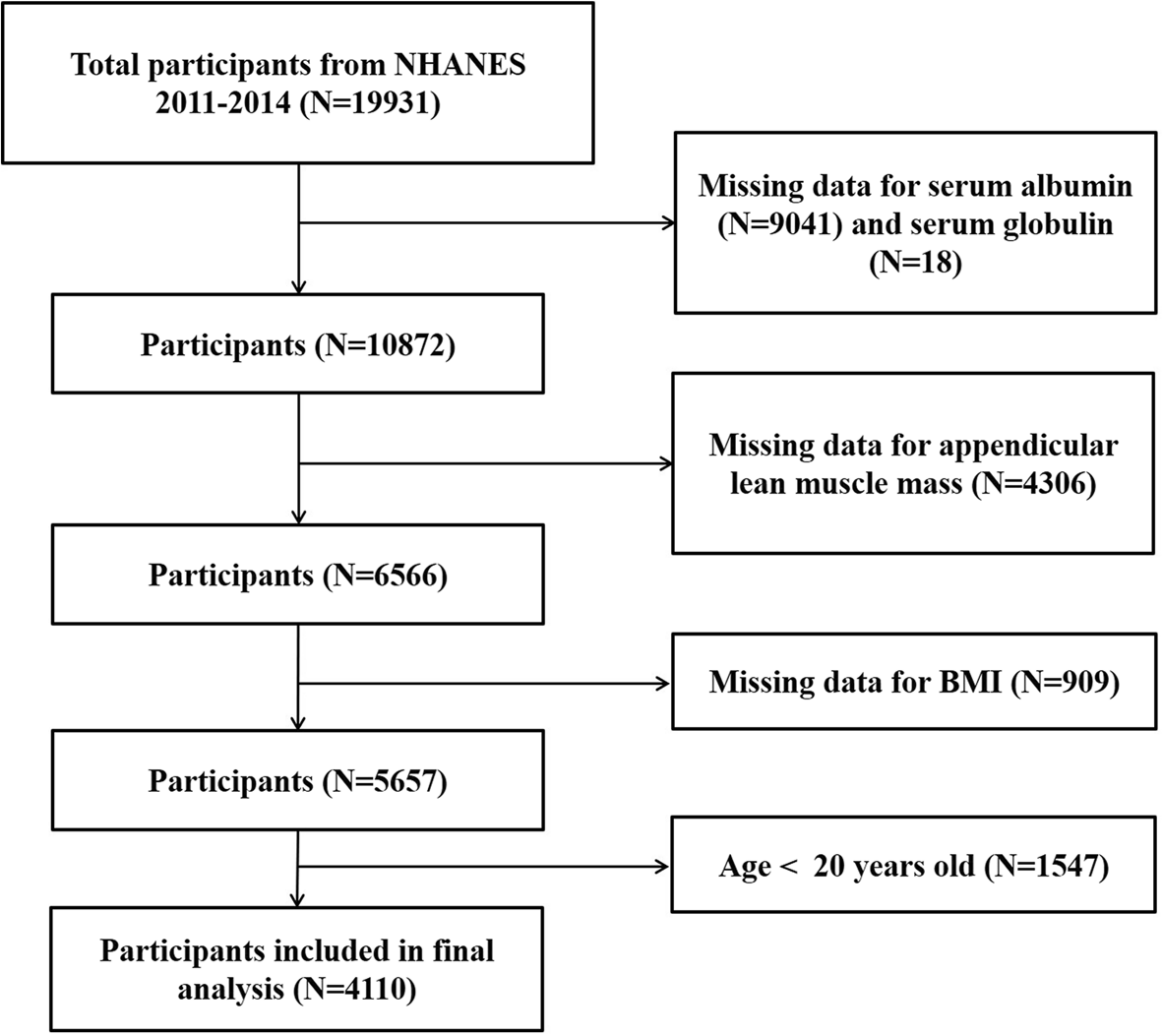
The flowchart of selection processes

We utilized data from 2 two-year cycles (2011–2012 and 2013–2014) of NHANES for analysis. Participants aged 20 years or older, with complete data of serum albumin, serum globulin, appendicular skeletal muscle mass (ASM) and BMI were included in this study. (The flowchart of selection processes is shown in Fig. 1.) Then data on serum albumin, serum globulin, ASM, BMI and potential confounders (sociodemographic characteristics, medical conditions, laboratory parameters) were extracted and aggregated.

### Study variables

#### Muscle mass

The body composition measurements were performed using whole-body dual-energy x-ray absorptiometry (DXA) exams (Hologic, Inc., Bedford, Massachusetts), which provided values for total and regional bone mineral content, fat mass and lean soft tissue mass. ASM was defined as the sum of the lean soft tissue mass of both arms and legs [15]. BMI was obtained from body measures. We further quantified muscle mass using appendicular skeletal muscle index (ASMI), calculated as the ratio of ASM and BMI [16]. Based on the Foundation for the National Institutes of Health recommendation, LMM was defined as ASMI < 0.789 for men and ASMI < 0.512 for women [16].

#### Serum albumin, globulin and AGR

The serum albumin (g/dL) and serum globulin (g/dL) were measured using a Beckman UniCel® DxC800 Synchron instrument [17]. The AGR was calculated as the albumin divided by the globulin.

#### Other covariates

The selection of confounding factors was based on literatures, and the following variables were considered as covariates. Demographic variables, including age (years), gender (man or woman), race (Hispanic, Non-Hispanic White, Non-Hispanic Black, others) were obtained from demographic data. The presence or absence of hypertension and diabetes were defined by participants’ self-report of the diagnosis by a doctor. The serum total calcium (mg/dL), phosphorus (mg/dL), triglycerides (mg/dL) and uric acid (mg/dL) were measured using a Beckman UniCel® DxC800 Synchron instrument. White blood cell counts (1000 cells/uL) and Hemoglobin (g/dL) were measured using the Beckman Coulter MAXM instrument [17]. The serum 25(OH)D (nmol/L) was quantified using ultra-high performance liquid chromatography-tandem mass spectrometry (UHPLC-MS/MS) [18]. The total cholesterol (mg/dL) in serum was determined using Roche/Hitachi Modular P Chemistry Analyzer [19]. (A summary of the reference range of laboratory parameters is demonstrated in Supplemental Table 1). A detailed description of the laboratory methodology, quality-control and quality-assurance protocols can be found on the NHANES website.

**Table 1:** Baseline characteristics of study participants

Characteristics	Man		*P* value	Woman		*P* value
	NMM (*n* = 1941)	LMM (*n* = 139)		NMM (*n* = 1875)	LMM (*n* = 155)	
Age (years)	38.9 (0.5)	44.3 (1.3)	< 0.001	39.7 (0.6)	45.3 (1.4)	< 0.001
BMI (kg/m^2^)	27.9 (0.2)	35.8 (1.0)	< 0.001	28.2 (0.2)	34.2 (0.8)	< 0.001
White blood cell count (1000 cells/uL)	7.0 (0.1)	8.2 (0.3)	0.001	7.3 (0.1)	7.7 (0.2)	0.092
Hemoglobin (g/dL)	15.2 (0.0)	15.0 (0.2)	0.271	13.3 (0.1)	13.3 (0.1)	0.933
Total calcium (mg/dL)	9.5 (0.0)	9.3 (0.1)	0.049	9.4 (0.0)	9.3 (0.0)	0.488
Phosphorus (mg/dL)	3.7 (0.0)	3.6 (0.0)	0.058	3.8 (0.0)	3.8 (0.0)	0.178
Uric acid (mg/dL)	6.0 (0.0)	6.2 (0.2)	0.204	4.6 (0.0)	4.7 (0.1)	0.394
Total Cholesterol (mg/dL)	192.3 (1.6)	194.0 (3.6)	0.698	193.9 (1.5)	200.3 (3.6)	0.130
Triglycerides (mg/dL)	163.9 (4.0)	214.5 (14.7)	0.003	128.3 (3.3)	154.0 (9.8)	0.018
Albumin (g/dL)	4.5 (0.0)	4.3 (0.0)	0.001	4.2 (0.0)	4.2 (0.0)	0.023
Globulin (g/dL)	2.7 (0.0)	2.8 (0.1)	0.007	2.8 (0.0)	3.0 (0.0)	0.001
AGR	1.7 (0.0)	1.6 (0.0)	< 0.001	1.5 (0.0)	1.4 (0.0)	< 0.001
25(OH)D (nmol/L)	64.6 (1.2)	55.2 (2.3)	< 0.001	69.3 (1.7)	64.7 (3.3)	0.151
ASM (kg)	27.5 (0.1)	26.3 (0.7)	0.093	18.6 (0.2)	16.2 (0.4)	< 0.001
ASMI	1.0 (0.0)	0.7 (0.0)	< 0.001	0.7 (0.0)	0.5 (0.0)	< 0.001
Race (%)			< 0.001			< 0.001
Hispanic	15.8	29.4		14.1	43.1	
Non-Hispanic White	64.6	60.1		65.3	44.6	
Non-Hispanic Black	11.1	2.9		12.3	5.9	
other	8.5	7.6		8.3	6.4	
Hypertension (%)			0.462			0.001
No	76.8	73.5		78.0	63.3	
Yes	23.2	26.5		22.0	36.7	
Diabetes (%)			0.019			< 0.001
No	95.1	88.5		94.9	84.3	
Yes	4.9	11.5		5.1	15.7	

*Abbreviations*: *NMM* Normal muscle mass, *LMM* Low muscle mass, *ASM* Appendicular skeletal muscle mass, *ASMI* Appendicular skeletal muscle index, *BMI* Body mass index, *AGR* Albumin to globulin ratio

The data were demonstrated as weighted mean (se) for continuous variables, and weighted percentage for categorical variables

### Statistical analysis

All statistical analyses were conducted by R 3.4.3 (https://www.r-project.org/) and EmpowerStats software (http://www.empowerstats. com), and P < 0.05 was considered statistically significant. NHANES sample weights was taken into account when calculated all estimates. Weighted linear regression model (for continuous variables) and weighted Chi-square test (for categorical variables) were performed to compare the baseline characteristics of the included participants. Weighted multiple linear regression analysis and smooth curve fittings were used to evaluate the relationship between albumin, globulin, AGR and ASMI. If there was a non-linear relationship, threshold effect analysis was performed using two-piecewise linear regression model.

## Results

There were 19,931 participants in the NHANES 2011–2014, 15,821 participants were excluded due to lack of sufficient data or younger than 20 years old, leaving 4110 participants for final analysis. As demonstrated in Table 1, the included participants were subclassified based on the presence or absence of LMM. There were 294 participants (man: 139, woman: 155) with LMM and 3816 participants (man: 1941, woman: 1875) with NMM. Compared to those with NMM, men and women with LMM were older, and had significantly greater prevalence of diabetes, higher BMI, serum globulin and triglycerides, lower serum albumin and AGR.

### Association between serum albumin and muscle mass

Table 2 demonstrates the association between serum albumin and muscle mass in three multivariate linear regression models. The serum albumin was positively correlated to muscle mass in all three models (model 1: β = 0.238, 95%CI:0.213–0.262; model 2: β = 0.070, 95%CI:0.051–0.090; model 3: β = 0.023, 95%CI:0.004–0.042). In subgroup analyses stratified by gender, there were a positive association in men, but a negative association in women. When stratified by muscle mass, a significantly positive association was observed only in NMM individuals.

**Table 2:** Relationship between albumin and ASMI

Outcome	Model 1		Model 2		Model 3	
	β (95%CI)	*P*-value	β (95%CI)	*P*-value	β (95%CI)	*P*-value
Albumin	0.238 (0.213, 0.262)	< 0.001	0.070 (0.051, 0.090)	< 0.001	0.023 (0.004, 0.042)	0.031
Stratified by gender						
Man	0.100 (0.077, 0.123)	< 0.001	0.074 (0.047, 0.010)	< 0.001	0.038 (0.012, 0.064)	0.010
Woman	0.058 (0.033, 0.082)	< 0.001	0.063 (0.036, 0.089)	< 0.001	-0.020 (-0.037, -0.002)	0.046
Stratified by MM						
NMM	0.232 (0.210, 0.255)	< 0.001	0.057 (0.036, 0.078)	< 0.001	0.023 (0.003, 0.043)	0.039
LMM	0.093 (0.010, 0.176)	0.037	0.003 (-0.025, 0.030)	0.844	-0.005 (-0.056, 0.047)	0.847

*ASMI* Appendicular skeletal muscle index, *MM* Muscle mass, *NMM* Normal muscle mass, *LMM* Low muscle mass

Model 1: no covariates were adjusted

Model 2: gender, age and race were adjusted

Model 3: gender, age, race, BMI, hypertension, diabetes, white blood cell count, hemoglobin, total calcium, phosphorus, uric acid, total cholesterol, triglycerides and 25[OH]D were adjusted

### Association between serum globulin and muscle mass

The effect sizes for the association between serum globulin and muscle mass are presented in Table 3. There were significantly negative associations between serum globulin and muscle mass in model 1 (β = -0.091, 95%CI: -0.110-(-0.073)) and model 2 (β = -0.038, 95%CI: -0.049-(-0.028)), but no significant association between them in model 3 (β = -0.010, 95%CI: -0.021–0.001). Stratified by gender and muscle mass, the significant association was observed only in men.

**Table 3:** Relationship between globulin and ASMI

Outcome	Model 1		Model 2		Model 3	
	β (95%CI)	*P*-value	β (95%CI)	*P*-value	β (95%CI)	*P*-value
Globulin	-0.091 (-0.110, -0.073)	< 0.001	-0.038 (-0.049, -0.028)	< 0.001	-0.010 (-0.021, 0.001)	0.059
Stratified by gender						
Man	-0.035 (-0.055, -0.015)	0.002	-0.040 (-0.061, -0.020)	0.001	-0.024 (-0.040, -0.008)	0.011
Woman	-0.032 (-0.042, -0.021)	< 0.001	-0.034 (-0.047, -0.021)	< 0.001	0.003 (-0.014, 0.019)	0.746
Stratified by MM						
NMM	-0.084 (-0. 104, -0.063)	< 0.001	-0.029 (-0.038, -0.019)	< 0.001	-0.010 (-0.019, 0.000)	0.075
LMM	-0.048 (-0.099, 0.003)	0.078	-0.000 (-0.020, 0.019)	0.978	-0.020 (-0.063, 0.023)	0.370

*ASMI* Appendicular skeletal muscle index, *MM* Muscle mass, *NMM* Normal muscle mass, *LMM* Low muscle mass

Model 1: no covariates were adjusted

Model 2: gender, age and race were adjusted

Model 3: gender, age, race, BMI, hypertension, diabetes, white blood cell count, hemoglobin, total calcium, phosphorus, uric acid, total cholesterol, triglycerides and 25[OH]D were adjusted

### Association between AGR and muscle mass

As show in Table 4, AGR was positively correlated to muscle mass in model 1 (β = 0.186, 95%CI:0.145–0.227) and model 2 (β = 0.064, 95%CI:0.042–0.087), but not in model 3 (β = 0.017, 95%CI: -0.001–0.034). Stratified by gender and muscle mass, the significant correlations retained only in men.

**Table 4:** Relationship between AGR and ASMI

Outcome	Model 1		Model 2		Model 3	
	β (95%CI)	*P*-value	β (95%CI)	*P*-value	β (95%CI)	*P*-value
AGR	0.186 (0.145, 0.227)	< 0.001	0.064 (0.042, 0.087)	< 0.001	0.017 (-0.001, 0.034)	0.060
Stratified by gender						
Man	0.072 (0.043, 0.100)	< 0.001	0.065 (0.035, 0.096)	< 0.001	0.032 (0.008, 0.055)	0.018
Woman	0.055 (0.038, 0.072)	< 0.001	0.059 (0.036, 0.083)	< 0.001	-0.006 (-0.028, 0.016)	0.583
Stratified by MM						
NMM	0.172 (0.131, 0.213)	< 0.001	0.048 (0.028, 0.068)	< 0.001	0.016 (-0.001, 0.032)	0.058
LMM	0.129 (0.057, 0.201)	0.001	0.002 (-0.032, 0.035)	0.925	0.022 (-0.050, 0.095)	0.551

*AGR* Albumin to globulin ratio, *ASMI* Appendicular skeletal muscle index, *MM* Muscle mass, *NMM* Normal muscle mass, *LMM* Low muscle mass

Model 1: no covariates were adjusted

Model 2: gender, age and race were adjusted

Model 3: gender, age, race, BMI, hypertension, diabetes, white blood cell count, hemoglobin, total calcium, phosphorus, uric acid, total cholesterol, triglycerides and 25[OH]D were adjusted

### Threshold effect analysis

**Fig. 2:**
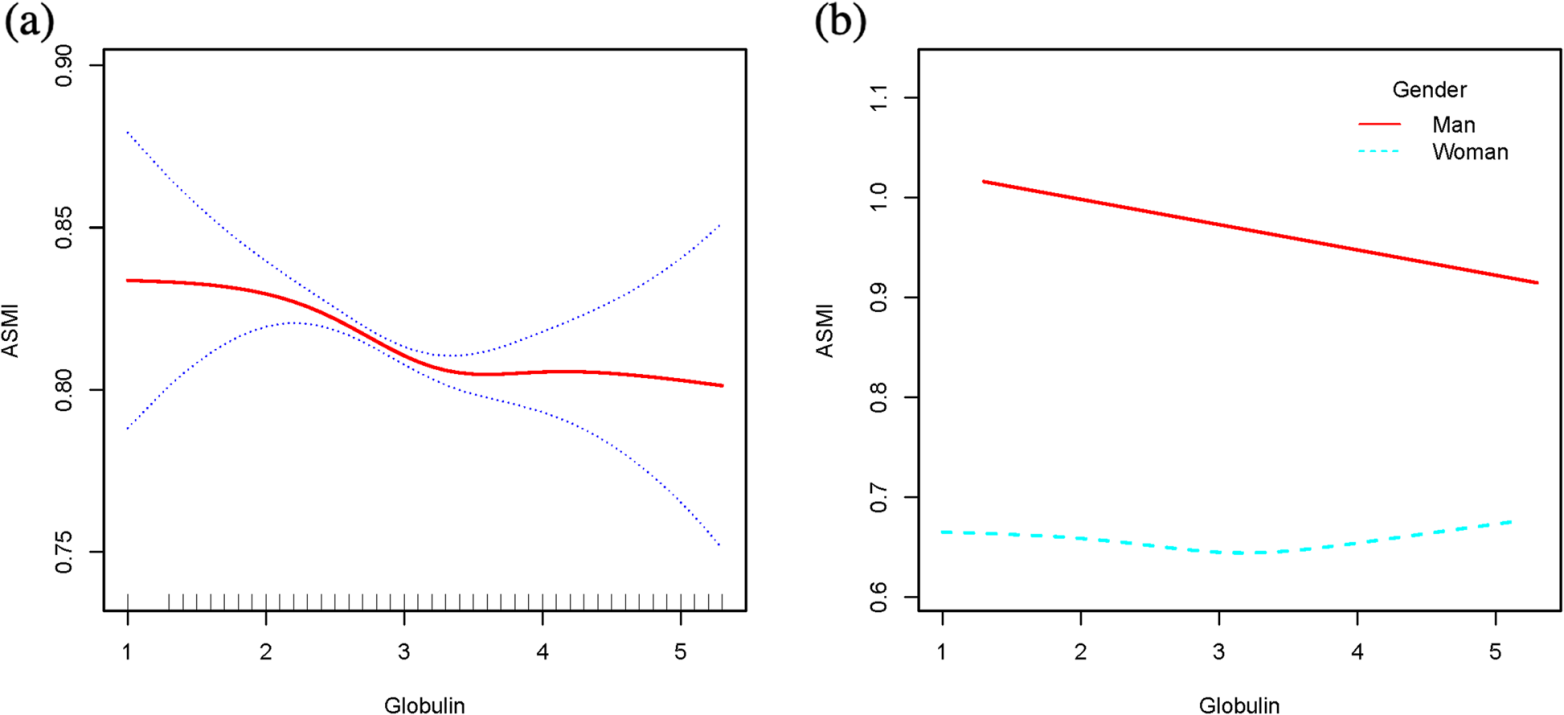
**a** The relationship between serum globulin and ASMI. The area between two blue dotted is expressed as 95% CI. Each point shows the magnitude of the globulin and is connected to form a continuous line. **b** The relationship between serum globulin and ASMI, stratified by sex

**Fig. 3:**
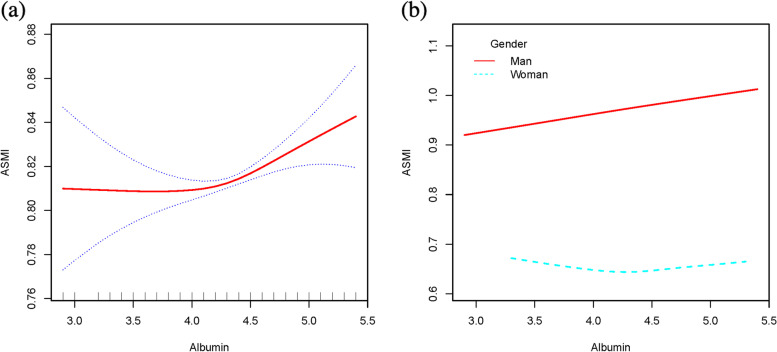
**a** The relationship between serum albumin and ASMI. The area between two blue dotted is expressed as 95% CI. Each point shows the magnitude of the albumin and is connected to form a continuous line. **b** The relationship between serum albumin and ASMI, stratified by sex

**Fig. 4:**
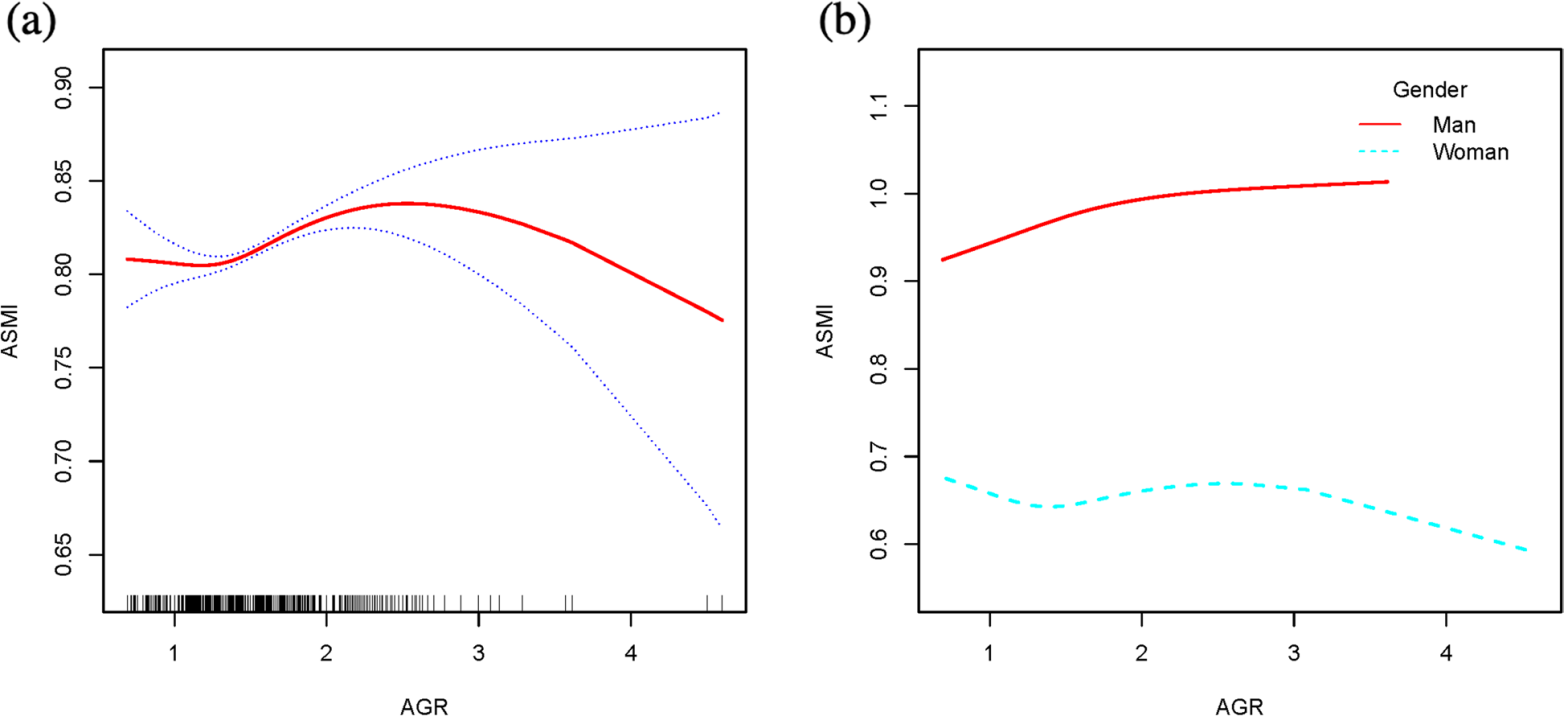
**a** The relationship between AGR and ASMI. The area between two blue dotted is expressed as 95% CI. Each point shows the magnitude of the AGR and is connected to form a continuous line. **b** The relationship between AGR and ASMI, stratified by sex

Generalized additive models and smooth curve fittings were performed to find the non-linear relationship between serum albumin, serum globulin, AGR and muscle mass (Figs. 2, 3 and 4). The results demonstrated linear relationships between globulin and muscle mass (Fig. 2b), albumin and muscle mass (Fig. 3b) in men, and non-linear relationships between albumin and muscle mass in women (Fig. 3b), AGR and muscle mass in men (Fig. 4b). Then threshold effect values of serum albumin in women and AGR in men were identified by using two-piecewise linear regression models (Table 5).

**Table 5:** Threshold effect analysis of albumin and AGR on ASMI

	Adjusted β(95%CI)	*P-*value
Woman		
Albumin < 4.3	-0.018 (-0.042, 0.006)	0.144
Albumin > 4.3	0.060 (0.032, 0.088)	< 0.001
Man		
AGR < 1.643	0.059 (0.024, 0.094)	0.001
AGR > 1.643	0.010 (-0.012, 0.031)	0.393

*AGR* Albumin to globulin ratio, *ASMI* Appendicular skeletal muscle index

Gender, age, race, BMI, hypertension, diabetes, white blood cell count, hemoglobin, total calcium, phosphorus, uric acid, total cholesterol, triglycerides, 25[OH]D were adjusted in the model

## Discussion

Various factors have been suggested to involve in the occurrence and development of muscle loss, chronic low-grade inflammation and malnutrition were considered to be important causative factors [8, 20]. Although several elevated inflammatory factors have been identified in LMM individuals, a biomarker with a clear link to muscle mass measurement is still lacking. The results of our study demonstrated a significantly higher serum globulin, and lower serum albumin and AGR in LMM individuals. Furthermore, albumin and AGR were positively correlated with muscle mass, globulin was negatively correlated with muscle mass in men.

There is a growing awareness that even health ageing cannot avoid an elevated inflammatory status, which is considered to be related with muscle loss and function impairment [21]. Several elevated inflammatory markers, including C-reactive protein (CRP), erythrocyte sedimentation rate (ESR), interleukin-6 (IL-6), and tumor necrosis factor-α (TNF-α), have been identified in patients with poor muscle strength and muscle mass [21, 22]. Serum globulin contains various proteins involving in inflammatory responses, such as complements, immunoglobulins, and acute phase response proteins, which has been recognized as a reliable indicator for inflammatory state [23]. However, the relationship between globulin and muscle mass has long been disregarded. Our study not only found a higher globulin level in individuals with LMM, but also identified a negative association between globulin and muscle mass in men. This finding further corroborated the role of inflammation in muscle loss.

Albumin, which has been commonly used for evaluating nutritional status, is currently regarded as an inflammatory marker. Previous studies demonstrated that it was a negative phase reactant that decreased with inflammation, irrespective of patients’ nutritional state [24, 25]. Some scholars hold the view that hypoalbuminemia might be related with inflammation rather than malnutrition [25]. Recently, several studies have been conducted to investigate the relationship between albumin and muscle function. Based on the results of a longitudinal study including 1320 older men and women, Schalk et al. suggested that low albumin levels, even within the normal limits, were independently associated with weaker muscle strength [26]. In another cross-sectional study, the authors reported that serum albumin levels were positively correlated with gait speed and handgrip strength [11]. The finding of our study further suggested a close relationship between albumin and muscle mass, which demonstrated a positive association between albumin and muscle mass in men. Two possible explanations were that, first, albumin reflected nutritional status, which was positively associated with muscle mass. Second, inflammatory mediators could not only promote albumin escape from blood capillaries, but also decrease the synthesis of albumin [24]. As both albumin and muscle mass were negatively associated with inflammation, there was no doubt that albumin would show a positive association with muscle mass.

The AGR, which takes both albumin and globulin into account, is a convenient serological indicator to identify serum protein abnormalities [27]. Several studies indicated that it could reflect not only a change in nutritional condition, but also a medium to long-term inflammatory status [27, 28]. Literatures reported AGR could serve as a diagnostic biomarker for infection diseases, or a prognostic marker for various types of cancer [29, 30]. However, few studies have investigated the relationship between AGR and muscle mass. Our study showed a positive association between AGR and muscle mass in men, which suggested it might also be a useful biomarker for assessing muscle mass.

Although evidences for sex difference in hemoglobulin, serum creatinine, serum immunoglobulin, serum albumin levels have been reported by several previous studies [31, 32]. There is a lack of knowledge about the effect of sex on the relationship between serum albumin, globulin and muscle mass. The present study demonstrated albumin, globulin, AGR were differentially associated with muscle mass between men and women. The inherent differences in hormone and inflammatory levels may partially explain the sex-specific outcomes [33], but further studies are still required to elucidate the underlying mechanisms.

### Limitation

There were some limitations in this study. Firstly, this was a cross-sectional study, so we could not establish a causal relationship between albumin, globulin, AGR and muscle mass. Secondly, some potential confounding factors, such as IL-6, TNF-α, CRP and ESR, were not available in this study. Finally, there were different adjustment methods for quantifying muscle mass, such as ASM/height^2^, ASM/weight, and ASM/BMI. It has been suggested that different adjustment methods may lead to different results in the relationship between muscle mass and clinical outcomes [34]. Therefore, consensus is urgently needed to standardize the adjustment method.

## Conclusion

The present study demonstrated a significantly higher serum globulin, and lower serum albumin and AGR in LMM individuals. The relationships between albumin, globulin, AGR and muscle mass were sex-specific, positive associations between albumin, AGR and muscle mass, and negative association between globulin and muscle mass were observed only in men. We speculate these indicators may be useful in assessing muscle mass in men.

## Supplementary Information

**Additional file 1: Supplemental table 1.**The summary of laboratory parameters

## Data Availability

The data are available from the corresponding author on reasonable request.

## Abbreviations

NHANES: National Health and Nutrition Examination Survey
AGR: Albumin to globulin ratio, BMI: body mass index
DXA: Dual-energy x-ray absorptiometry
ASM: Appendicular skeletal muscle mass
ASMI: Appendicular skeletal muscle index
LMM: Low muscle mass
NMM: Normal muscle mass
CRP: C-reactive protein
ESR: Erythrocyte sedimentation rate
IL-6: Interleukin-6
TNF-α: Tumor necrosis factor-α
