# Supplementary material for: Associations between albumin, globulin, albumin to globulin ratio and muscle mass in adults: results from the national health and nutrition examination survey 2011–2014
Source: BMC Geriatr. 2022 May 2;22:383. doi: 10.1186/s12877-022-03094-4 (PMC9059414; doi:10.1186/s12877-022-03094-4)
Supplement: Supplementary file 1 — Additional file 1: Supplemental table 1.The summary of laboratory parameters [file 12877_2022_3094_MOESM1_ESM.docx]

| Supplemental table 1. The summary of laboratory parameters. | | |
| --- | --- | --- |
| Parameters | Range of value | Reference Range |
| Albumin (g/dL) | 2.1-5.5 | 3.7-4.7 |
| Globulin (g/dL) | 0.7-5.9 | NA |
| Total protein (g/dL) | 4.7-10.2 | 6.4-7.7 |
| Total calcium (mg/dL) | 6.5-11.3 | 8.5-10.5 |
| Phosphorus (mg/dL) | 1.6-7.2 | 2.6-4.4 |
| Triglycerides (mg/dL) | 12-3187 | 0-1000 |
| Uric acid (mg/dL) |  |  |
| Male | 0.4-11.3 | 3.6-8.4 |
| Female | 0.4-11.3 | 2.9-7.5 |
| White blood cell count (1000 cells/uL) | 1.7-54.7 | 3.9-11.8 |
| Hemoglobin (g/dL) | 6.1-19.6 | 13.1-17.5 |
| 25(OH)D (nmol/L) | 7.56-375 | ≥ 50 |
| Total cholesterol (mg/dL) | 59-523 | 100-200 |
